# Human Bronchial Epithelial Cells Inhibit *Aspergillus fumigatus* Germination of Extracellular Conidia *via* FleA Recognition

**DOI:** 10.1038/s41598-018-33902-0

**Published:** 2018-10-24

**Authors:** Nicolas Richard, Léa Marti, Annabelle Varrot, Loïc Guillot, Juliette Guitard, Christophe Hennequin, Anne Imberty, Harriet Corvol, Michel Chignard, Viviane Balloy

**Affiliations:** 1 Sorbonne Université, UPMC Univ. Paris 06, Inserm, Centre de Recherche Saint-Antoine Paris, Paris, France; 2 Université Grenoble Alpes, CNRS, CERMAV, 38000 Grenoble, France; 3 Service de Parasitologie-Mycologie, Hôpital St Antoine, AP-HP, Paris, France; 4 Pneumologie Pédiatrique, AP-HP, Hôpital Trousseau, Paris, France

## Abstract

*Aspergillus fumigatus* is an environmental filamentous fungus that may act as an opportunistic pathogen causing a variety of diseases, including asthma or allergic bronchopulmonary aspergillosis, and infection, ranging from asymptomatic colonization to invasive pulmonary form, especially in immunocompromised patients. This fungus is characterized by different morphotypes including conidia which are the infective propagules able to germinate into hyphae. Due to their small size (2–3 µm), conidia released in the air can reach the lower respiratory tract. The objective of this study was to characterize the interactions between conidia and bronchial epithelial cells. To this end, we studied the role of bronchial epithelial cells, *i*.*e*., the BEAS-2B cell line and human primary cells, in conidial germination of a laboratory strain and three clinical strains of *A*. *fumigatus*. Microscopic observations and galactomannan measurements demonstrated that contact between epithelial cells and conidia leads to the inhibition of conidia germination. We demonstrated that this fungistatic process is not associated with the release of any soluble components nor internalization by the epithelial cells. We highlight that this antifungal process involves the phosphoinositide 3-kinase pathway on the host cellular side and the lectin FleA on the fungal side. Collectively, our results show that bronchial epithelial cells attenuate fungal virulence by inhibiting germination of extracellular conidia, thus preventing the morphological change from conidia to filaments, which is responsible for tissue invasion.

## Introduction

*Aspergillus fumigatus* is an environmental filamentous fungus that can act as an opportunistic pathogen and cause a variety of lung diseases, including invasive diseases, chronic diseases, and allergic reactions^1^. Although the invasive aspergillosis developed by immunocompromised patients is the most severe clinical form, the prevalence is much higher for the chronic and allergic forms (1).

The first site of infection is the respiratory system. Owing to their small size (2–3 µm), the conidia (the infectious morphotype of *A*. *fumigatus* released in the air) can reach the lower respiratory tract^2^. Under certain conditions, such as immunosuppression, or in the presence of underlying pulmonary diseases such as allergic asthma, cystic fibrosis (CF), and chronic obstructive pulmonary disease (COPD), *A*. *fumigatus* can persist in airways and become infectious^3^. However, conidia inhaled by healthy people with functional immune systems and normal airway function are rapidly cleared. Most of the inhaled conidia are eliminated mechanically by coughing and sneezing, allowing the removal of inhaled conidia trapped in the mucus and transported by ciliated cells. Conidia that succeed in crossing this barrier interact first with the airway epithelium. The bronchial epithelium interacting with conidia and filaments triggers an innate immune response, and thus participates directly or indirectly in the clearance of *A*. *fumigatus* from the lungs^4,5^.

Conidia have been shown to adhere to the epithelial cells and extracellular matrix exposed in airways of patients at risk^6–8^. Afterwards, they can be internalized by the respiratory epithelial cells^9^, where some survive and escape from immune cells^10,11^. In a previous study, we demonstrated that bronchial epithelial cells can also recognize and be activated by germinating conidia and hyphae to produce IL-8, a chemokine involved in the recruitment of polymorphonuclear cells, the key cells in the immune response against both morphotypes of *A*. *fumigatus*^12^.

Nonetheless, mechanisms of conidia clearance from the lungs are not completely understood. Lectins, expressed by host cells or by fungi, are involved in the host-pathogen interactions in recognizing carbohydrates. Some are well described, such as dectin-1, a C-type lectin expressed by macrophages, that recognizes β-1-3 glucan on the surface of *A*. *fumigatus* germ tubes and stimulates TNF-alpha production^13^. Lung pathogens, such as *Pseudomonas aeruginosa*, *Burkholderia cenocepacia*, and *Aspergillus fumigatus*, expressed lectins used as adhesins to interact with host glycoproteins and/or glycolipids^14–16^. The FleA lectin (also called AFL) has been identified to be expressed by resting *A*. *fumigatus* conidia and to bind mucins of the airway mucus^17^. Moreover, this lectin has been shown to interact with fucosylated structures and to be involved in the interaction of *A*. *fumigatus* and bronchial epithelial cells^16^.

Although resident macrophages and recruited innate immune phagocytes are crucial in the removal of *A*. *fumigatus*^12,18^, very few studies have investigated the potential antifungal activity of respiratory epithelial cells. In this study, we investigated the role of bronchial epithelial cells in the clearance of *A*. *fumigatus* and highlighted their capacity to impact the germination of conidia *via* the PI3-kinase pathway and the interaction with the lectin FleA.

## Results

### Bronchial epithelial cells inhibit the filament formation of *A*. *fumigatus*

**Figure 1:**
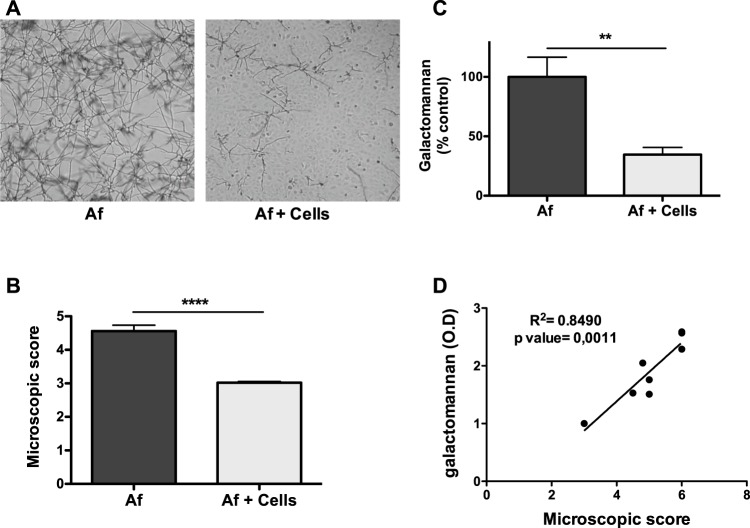
Effect of epithelial cell line (BEAS-2B) on filament formation when co-cultured with *A*. *fumigatus* conidia. (**A**) Filament formation of *A*. *fumigatus* (DAL strain) conidia cultured for 15 h with or without BEAS-2B cells (magnification, 40×). (**B**) Scoring of *A*. *fumigatus* filament formation when cultured with or without BEAS-2B (arbitrary units). (**C**) Galactomannan released (% of the control: *A*. *fumigatus* without cells). (**D**) Correlation between microscopic score and galactomannan measurement (Pearson’s test). Data are presented as mean ± SEM; n = 7 independent experiments performed in triplicate. **p < 0.01; ****p < 0.0001 (Student’s *t*-test); Af, *A*. *fumigatus*.

BEAS-2B cells were infected for 15 h with 10^3^ DAL strain conidia, and in parallel, conidia were incubated under the same conditions without epithelial cells. Optical microscopy examinations showed a reduction in filament formation following the incubation of conidia with epithelial cells compared with that of the fungus incubated in the medium alone (Fig. 1A). Two quantitative approaches were used: scoring the filament formation observed by microscopy and measuring the amount of galactomannan, a polysaccharide released in the supernatant by *A*. *fumigatus* during growth. The microscopic score showed a significant decrease from 4.56 ± 1.18 to 3.02 ± 0.04 when comparing filament formation by conidia incubated without or with bronchial epithelial cells, respectively (Fig. 1B). Similarly, the galactomannan index declined significantly from 100.0% ± 16.6 to 34.6% ± 6.1, respectively (Fig. 1C). These results show that epithelial cells are able to inhibit *A*. *fumigatus* filament formation. We performed multiple measurements to confirm that the microscopic score and galactomannan measurements were significantly correlated (n = 7; r = 0.8490; p = 0.001) (Fig. 1D). This result validates the use of the galactomannan assay to quantify filament formation.

### Validation of the antifungal activity using clinically isolated strains and primary bronchial epithelial cells

**Figure 2:**
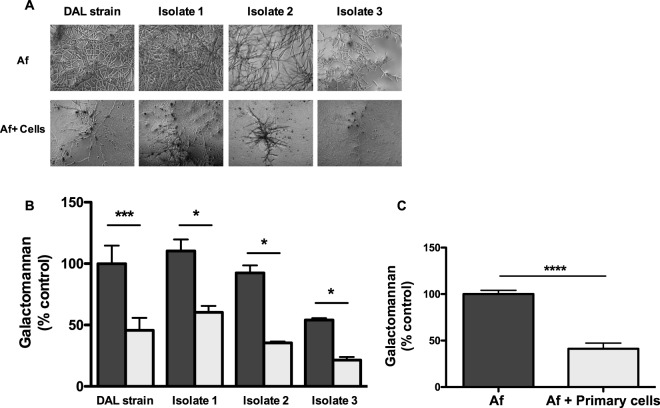
Filament formation by *A*. *fumigatus* laboratory strain (DAL) and clinical isolates in the presence or absence of the BEAS-2B epithelial cell line or primary bronchial epithelial cells. (**A**) Microscopic observations of filament formation from conidia of different origins cultured with and without BEAS-2B cells for 15 h (magnification, 40×). (**B**) Galactomannan released by laboratory (DAL) and three clinical isolates cultured with (Grey bars) and without (Black bars) BEAS-2B cells (% of the control: DAL strain of *A*. *fumigatus* without epithelial cells). Data are presented as mean ± SEM of 3 different experiments performed in triplicate. *p < 0.05; **p < 0.01 (Student’s *t*-test); Af, *A*. *fumigatus*. (**C**) Primary bronchial epithelial cells (Primary cells) inhibited the filament formation of *A*. *fumigatus*. Galactomannan released by *A*. *fumigatus* (DAL strain) cultured with or without primary epithelial cells for 15 h (% of the control: *A*. *fumigatus* without cells). Data are presented as mean ± SEM, n = 6 independent experiments performed in triplicate. ****p < 0.0001 (Student’s *t*-test); Af, *A*. *fumigatus*.

To confirm our results, we analyzed the antifungal activity of the bronchial epithelial cell line against *A*. *fumigatus* clinical strains isolated from patients with COPD or CF. We tested three different clinical strains under the above-described conditions. Noticeable inhibition of filament formation was microscopically observed for all strains when conidia were incubated with epithelial cells (Fig. 2A). Inhibition was significant for all the strains as indicated by the galactomannan index (47 ± 17; 45 ± 0.1; 61 ± 4 and 61 ± 4% inhibition for DAL strain, isolate 1, 2 and 3, respectively, Fig. 2B). These observations demonstrated that the bronchial epithelial cell line also inhibited the filament formation of *A*. *fumigatus* clinical strains.

BEAS-2B cells were isolated from human bronchial epithelium and then immortalized. As this may induce a bias, we extended our experiments to primary bronchial epithelial cells, isolated by Epithelix Sarl from six healthy donors. Primary epithelial cells cultured in monolayers were infected under the conditions described above. Filament formation of DAL strain conidia significantly decreased when they were incubated with primary epithelial cells from 100.0 ± 4.0 to 41.2 ± 6.1%, measured as the galactomannan index (Fig. 2C). These results confirm the findings obtained with the BEAS-2B epithelial cell line.

### Characterization of the mechanisms underlying antifungal activity

**Figure 3:**
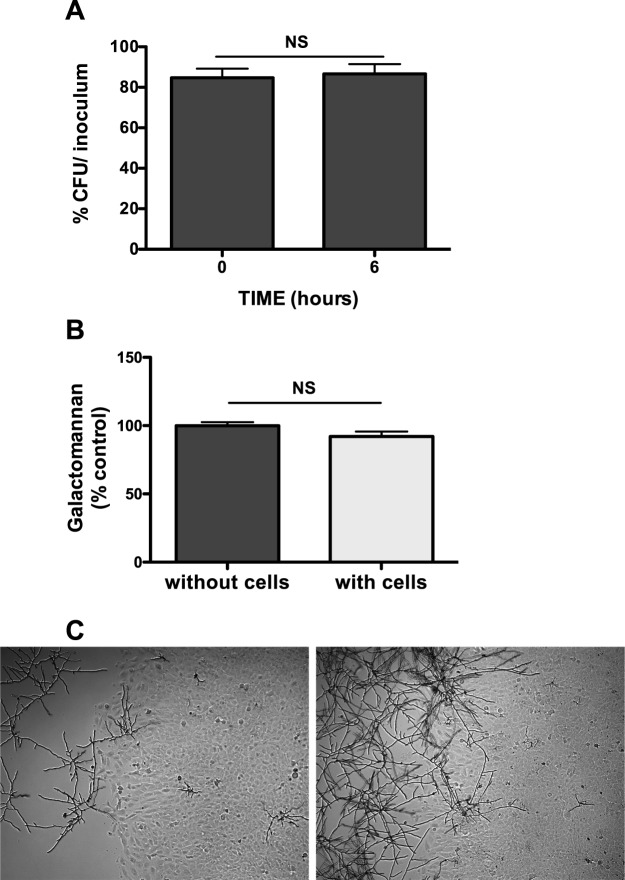
Characterization of the antifungal activity of bronchial epithelial cells. (**A**) Characterization of the antifungal activity of epithelial cells as fungistatic. Percentage of CFU relative to the inoculum at 0 and 6 hours after contact with BEAS-2B cells. Data are presented as mean ± SEM, n = 3 independent experiments performed in triplicate. NS, not significant (Student’s *t*-test). (**B**) Measurement of galactomannan release when conidia were incubated in inserts placed in wells with or without BEAS-2B cells (% of the control: *A*. *fumigatus* without cells). Data are presented as mean ± SEM, n = 3 independent experiments performed in triplicate. NS, not significant (Student’s *t*-test). (**C**) A lane was scratched in the BEAS-2B monolayer cultures and 10^3^ conidia (left) or 10^4^ (right) conidia were added for a 15-h incubation. Two representative images obtained from five independent experiments. Magnification, 40×.

To characterize the observed antifungal activity, we first evaluated the fungicidal or fungistatic effect. To this end, we measured the viability of the conidia after inoculation into BEAS-2B cells for 6 h. Conidia of the inoculum (0 h) collected 6 h after infection were processed for CFU determination. The proportion of CFUs did not differ significantly between the inoculum (84.7 ± 7.8%) and the sample from 6 h following infection (86.6 ± 8.4%), indicating that the epithelial cells inhibited filament formation without killing the conidia (Fig. 3A). These results show that the antifungal activity of the bronchial epithelial cells was fungistatic and not fungicidal.

We then determined the involvement of a putative soluble compound responsible for the fungistatic activity of the epithelial cells using two different approaches. The first was to prevent direct contact between conidia and epithelial cells using transwells. Filament formation of the DAL strain conidia placed in the inserts was not significantly different with or without epithelial cells overlaying the plate wells. These observations were confirmed by measuring the galactomannan index (92.0 ± 6.4% in presence and 100.0% ± 4.6 in the absence of cells, Fig. 3B). This result, indicated the absence of a soluble antifungal compound, and so a second approach was used. Thus, a lane was scratched in the confluent epithelial cell monolayers to expose the plastic surface in one part of the wells. Following incubation with 10^3^ conidia for 15 h, we observed filament growth under the microscope and noticed that although the growth of conidia was inhibited on the cell monolayer, the conidia formed filaments on the exposed plastic surface. Moreover, we did not observe a gradient of fungal growth at the edges of the cell monolayer, which would indicate that an antifungal compound was released from the cells (Fig. 3C). The differences observed led us to conclude that no soluble antifungal component was released.

### Identification of kinase pathways underlying the antifungal activity of epithelial cells

**Figure 4:**
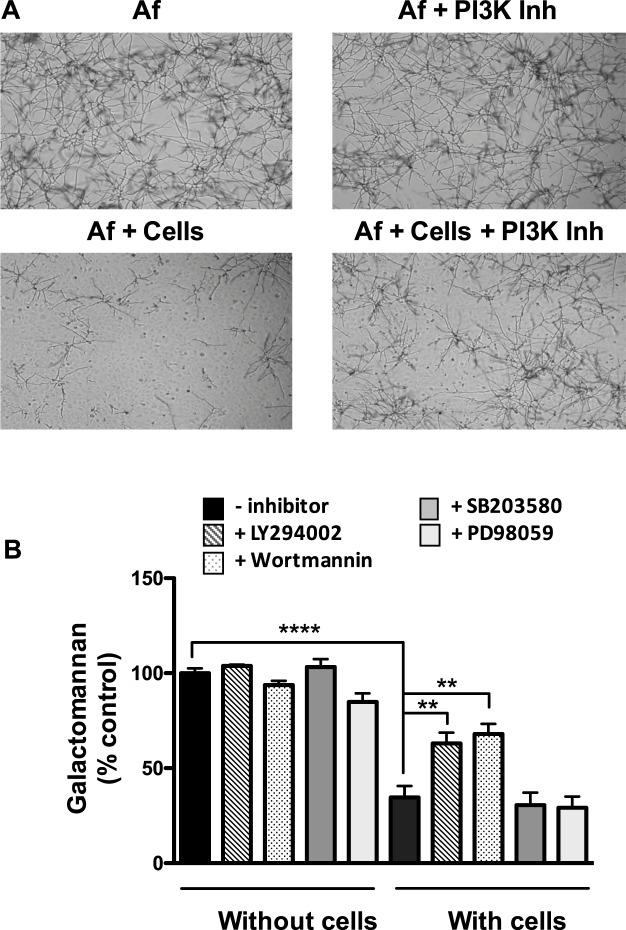
Involvement of PI3-kinase in the antifungal activity of bronchial epithelial cells against *A*. *fumigatus*. (**A**) A. *fumigatus* cultured with or without BEAS-2B epithelial cells, in the presence or the absence of LY294002, a PI3K inhibitor (magnification, 40×). (**B**) Galactomannan released (% of the control: *A*. *fumigatus* without cells and without inhibitors) with or without epithelial cells, and with or without inhibitors of PI3-kinase (LY294002 and wortmannin), p38 MAPK (SB203580), and ERK1/2 (PD98059). Data are presented as mean ± SEM, n = 3 independent experiments performed in triplicate. ***p < 0.001; **p < 0.01; NS, not significant (ANOVA, corrected for multiple testing using Bonferroni’s method); Af, *A*. *fumigatus*; Inh, inhibitor.

Kinases play a key role in the induction of the inflammatory cytokine response of activated bronchial epithelial cells, as we demonstrated in a previous study^12^. This prompted us to investigate whether PI3-kinase, p38 MAPK, and ERK1/2 were involved in the antifungal activity. BEAS-2B cells were pre-incubated for 1 h with LY294002 and wortmannin, two inhibitors targeting PI3-kinase, and SB203580 and PD98059 targeting p38 MAPK and ERK1/2, respectively. All inhibitors were used at previously reported concentrations^12^, and DMSO, the solvent, was used as the control. Afterward, 10^3^ conidia were added into each well and incubated at 37 °C for 15 h. Inhibitors and DMSO were also added to conidia alone to study their direct impact on the filament formation. No differences in filament formation were microscopically observed between conidia alone incubated with or without the inhibitors. Nonetheless, when comparing filament formation in the presence of cells, we confirmed that filament formation was decreased in the presence of epithelial cells and interestingly, we observed that this decrease was inhibited by the presence of PI3-kinase inhibitors (Fig. 4A) and not by p38 MAPK and ERK1/2 inhibitors (data not shown). These observations were quantified and confirmed using the galactomannan amount measured as a % of the control (fungus growth without cells and without kinase inhibitors). We showed that p38 MAPK, ERK1/2, and PI3-kinase inhibitors (LY294002 and wortmannin) did not exert a significant direct inhibitory effect on galactomannan release (103.1% ± 7.2; 84.9% ± 7.9; 103.8% ± 1.2; 93.8% ± 3.9, respectively) by fungus incubated in the absence of cells compared to the control (100.0% ± 4.3) (Fig. 4B). When incubated with cells, we observed that the galactomannan decrease (34.6% ± 6.1 of the control) was restored to 63.0% ± 5.7 and 68.0% ± 9.1 of the control only when cells were incubated with LY294002 and wortmannin, respectively. By contrast, the inhibitors of the other two kinases, p38 MAPK and ERK1/2, had no effect (Fig. 4B). These galactomannan data and microscopic observations demonstrate that the PI3-kinase pathway is involved in the antifungal activity of bronchial epithelial cells against *A*. *fumigatus*.

### Role of PI3-kinase in the antifungal activity of bronchial epithelial cells

**Figure 5:**
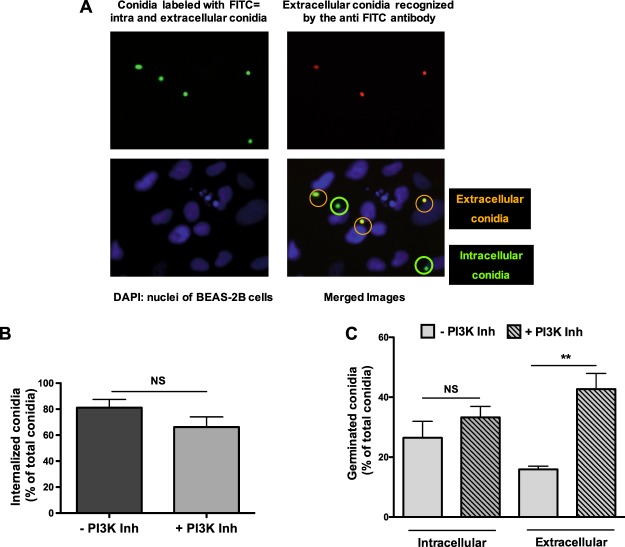
Role of PI3-kinase in the antifungal activity of epithelial cells. (**A**) Epifluorescence microscopic observations of FITC-labelled conidia incubated with BEAS-2B epithelial cells for 6 h. A combination of anti-FITC antibody and secondary antibody labelled with red AlexaFluor 568 was then added to labelled external conidia. Cell nuclei were stained with DAPI. One representative of 15 experiments is presented (magnification, 40×). (**B**) Percentage of internalized conidia in cells incubated with (+) or without (−) LY294002, the PI3-kinase inhibitor. Data are presented as mean ± SEM, n = 3 independent experiments performed in triplicate. NS, not significant (Student’s *t*-test). (**C**) Percentage of intra- or extracellular germinated conidia in the presence (+) or the absence (−) of LY294002, a PI3-kinase inhibitor. Data are presented as mean ± SEM, n = 4 independent experiments performed in triplicate. **p < 0.01; NS, not significant (ANOVA, corrected for multiple testing using Bonferroni’s method); Inh, inhibitor.

It has been shown that bronchial epithelial cells are able to internalize *A*. *fumigatus* conidia in acidic organelles^11^. As PI3-kinase is known to be involved in the internalization of bacteria by epithelial cells^19,20^, we hypothesized that the antifungal activity might be associated with the conidial internalization by epithelial cells. As shown in Fig. 5A, FITC-labeled conidia were incubated with BEAS-2B cells for 6 h. Then, extracellular conidia were detected using an anti-FITC antibody and a secondary antibody labeled with red AlexaFluor 568. Epifluorescence microscopic analyses (Fig. 5B) demonstrated no significant differences in the extent of conidia internalization by the epithelial cells when incubated with or without the PI3-kinase inhibitor LY294002 (66.2% ± 7.9 and 81.1% ± 6.4, respectively). These results show that we could not relate the antifungal activity to conidia internalization. Therefore, we determined whether the antifungal activity of epithelial cells was directed against extra- or intracellular conidia. For this, we counted the conidia that had germinated inside and outside the epithelial cells, in the presence or absence of a PI3-kinase inhibitor (LY294002). We observed that although the incubation of cells with the PI3-kinase inhibitor did not modify the rate of germination of intracellular conidia, the rate of germination of extracellular conidia significantly increased from 15.9% ± 2.1 to 42.7% ± 10.4 (Fig. 5C). These results suggest that the presence of PI3-kinase inhibitors restores the germination of only extracellular conidia. We concluded that the observed decrease in filament formation was caused by a decrease in the germination of extracellular conidia. Since free conidia were eliminated by washing before labeling, this inhibitory effect seems to concern only conidia attached to the surface of bronchial epithelial cells.

### Involvement of the lectin FleA in the antifungal activity of bronchial epithelial cells

We previously demonstrated that the purified lectin FleA, expressed by *A*. *fumigatus*, interacts with bronchial epithelial cells. Indeed, recombinant purified FleA induced bronchial epithelial cell activation (16). Thus, we hypothesized that FleA may participate in the interaction between epithelial cells and conidia under our experimental conditions and may be involved in the observed antifungal activity.

**Figure 6:**
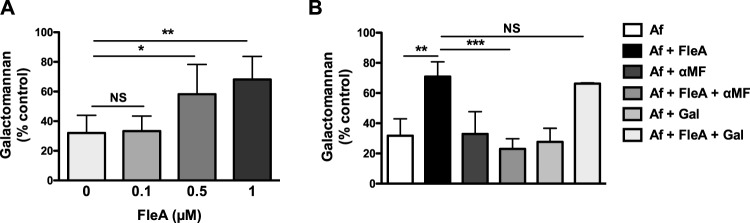
Effect of the lectin FleA on the antifungal activity of epithelial cells. (**A**) Galactomannan released by *A*. *fumigatus* cultured with epithelial cells in the presence of the lectin, FleA (0, 0.1, 0.5, and 1 µM) (% of the control: *A*. *fumigatus* without cells and without FleA). Each histogram is the mean ± SEM of three experiments performed in triplicate. *p < 0.05; **p < 0.01 (ANOVA test). (**B**) Galactomannan released by *A*. *fumigatus* cultured with epithelial cells preincubated with or without the lectin, FleA (1 µM) and methyl-α-L-fucopyranoside (αMF) (1 mM) or D-galactose (Gal) (1 mM). Data are presented as mean ± SEM obtained with 3 clinical strains in triplicate. ***p < 0.001, **p < 0.01 (ANOVA test).

Epithelial cells were incubated with different concentrations of purified FleA (0.1, 0.5, and 1 µM) 1 h before and during the infection by *A*. *fumigatus* for 15 h. We observed that FleA, at 0.5 and 1 µM, reversed the inhibition of filament growth in the presence of epithelial cells, as shown by the higher production of galactomannan (Fig. 6A). Under these conditions, no effect of FleA alone was observed on the filament formation of *A*. *fumigatus* when incubated without epithelial cells (data not shown). As FleA is reported to have a high affinity for fucose and fucose-containing oligosaccharides^16,21^ and, so, we incubated epithelial cells with FleA and methyl-α-L-fucopyranoside (αMF), a high affinity derivative of fucose, 1 h before and during the infection by *A*. *fumigatus* for 15 h. D-galactose, a monosaccharide not recognized by FleA, was also assayed as negative control. We observed that masking FleA by αMF restored the cellular antifungal activity whereas D-galactose did not have any effect (Fig. 6B). To better understand the role of FleA in its interaction with cells, we then examined if its presence could modify the conidial adhesion to epithelial cells. We observed that adhesion of conidia at 2 and 4 hours (97.5% ± 0.8 and 99.2% ± 0.4, respectively) was not significantly modified by the addition of 1 µM FleA (91.9% ± 5.8 and 98% ± 1.3, respectively).

These observations led us to conclude that FleA is a fungal compound that interacts specifically with bronchial epithelial cells and that is involved in the antifungal activity but not in the adhesion of conidia to the cells.

## Discussion

*Aspergillus fumigatus* is an environmental pathogen with the capacity to cause life-threatening diseases under specific conditions, such as an immune dysfunction of the host. The prerequisite for the development of pathogenic conditions is the germination of conidia, the infective propagules of *A*. *fumigatus*. Resting conidia represent the quiescent morphotype of *A*. *fumigatus* and are dispersed by air (1).

Most conidia following their entry into the lung, are rapidly phagocytosed and eliminated by alveolar macrophages^22^. Then, migrating polymorphonuclear neutrophils enter the stage and, in turn, participate in the elimination of conidia and filaments^23^. Nonetheless, previous studies also described the involvement of non-professional phagocytes, such as platelets, endothelial cells^24^, and epithelial cells^4,5^ in the antifungal host response. For example, Perkhofer *et al*.^25^ showed that *A*. *fumigatus* conidia activate platelets that can prevent fungal galactomannan release and hyphal elongation, whereas Paris *et al*.^26^ showed that conidia can be internalized by endothelial cells.

It is also well recognized that inhaled resting conidia interact first with bronchial epithelial cells. This first step is crucial as it could lead to persistence of *Aspergillus* and development of infections. Our present results demonstrate the ability of bronchial epithelial cells to attenuate the virulence of *A*. *fumigatus* by reducing conidial germination and filament formation, the invasive *A*. *fumigatus* morphotype. Thus, we microscopically observed a considerable inhibition of filament formation following the incubation of *A*. *fumigatus* with epithelial cells, compared with that in the absence of epithelial cells. These microscopic observations obtained with the bronchial epithelial cell line (BEAS-2B) were confirmed by the decrease in fungal galactomannan release. Furthermore, this bronchial epithelial cell line showed the same antifungal activity against strains freshly isolated from patients with COPD or CF. Moreover, human primary bronchial epithelial cells were shown to inhibit filament formation as well.

Previous studies investigating the potential antimicrobial activity of bronchial epithelial cells demonstrated that these cells secrete compounds that exert antifungal activity^27^. Alekseeva *et al*.^28^ reported an increase in the secretion of human beta defensins hBD-2 and hBD-9 by bronchial epithelial cells following their exposure to conidia. Secretome analysis of bronchial epithelial cells showed they interacted with *A*. *fumigatus* hyphae by releasing lysosomal enzymes, such as *N*-acetyl-β-D-glucosaminidase^29^. However, in this study, we demonstrated that the inhibition of the filament formation was not associated with the secretion of an antifungal compound. Furthermore, we determined that the antifungal activity of the bronchial epithelial cells was fungistatic and dependent on PI3-kinase pathway activation. Indeed, inhibition of the PI3-kinase pathway reduced the epithelial fungistatic activity, although it was only by 50%. In a previous study, we used a similar concentration of LY294002 (30 µM) that completely inhibited IL-8 production by BEAS-2B cells stimulated with *A*. *fumigatus* (12). It could be inferred that in the present study, PI3-kinase was completely inhibited by 30 µM LY294002, consequently, suggesting that along with PI3-kinase, another pathway was most likely involved in the fungistatic effect. PI3-kinase is an intracellular signal transducer enzyme that has been shown to be associated with diverse cellular functions, such as cell growth, proliferation, differentiation, motility, survival, and intracellular trafficking^30–32^. Furthermore, several studies have reported that the PI3-kinase signaling pathway is involved in bacterial invasion into host cells^19,33^. Conidia were shown to be engulfed by alveolar epithelial A549 cells *in vitro*, and the germination of the internalized conidia was shown to be delayed compared with that of the extracellular conidia^7,10,34^. To understand if the antifungal activity was related to the conidia internalization in our experimental conditions, we compared the internalization of conidia by bronchial epithelial cells in the presence or absence of a PI3-kinase inhibitor. We confirmed that epithelial cells can internalize conidia. However, neither the internalization of conidia nor the germination of the internalized conidia was modified following the inhibition of PI3-kinase. In contrast, PI3-kinase inhibition was shown to increase germination of the extracellular conidia. Taken together, our results showed that the effects of PI3-kinase on antifungal activity were primarily directed against the extracellular conidia attached to the surface of bronchial epithelial cells.

Whereas it is well described that polysaccharides (e.g., galactofuranose and galactosaminogalactan), expressed by swollen conidia and germlings, are required to interact with the host cell surface^35^, little is known about resting conidia ligands involved in the interaction with cells. Nonetheless a lectin, FleA, localized on the surface of *A*. *fumigatus* resting conidia, has been identified as a pathogen-associated molecular pattern recognized by alveolar macrophages^17^. FleA has been shown, by us and others^16,17,36^, to induce an immune response from the epithelial cells, which helps protect the host in immunocompetent situations. In the present study, we showed that FleA is involved in the bronchial epithelial antifungal activity but not in the adhesion of conidia to the cells, suggesting that other receptors are involved in the adhesion step. To explain that the addition of recombinant FleA reversed the fungistatic activity of epithelial cells whereas FleA present on conidia, would be involved in the failure of conidial germination, one can refer to the fibril hypothesis. According to this concept, soluble short oligosaccharides recognize a single receptor without inducing an immune response, whereas fibrillar polysaccharides bind to several receptors leading to increased avidity, receptor clustering, triggering of signaling events^37^. Interestingly, in this line, it has been described that another molecule expressed by conidia, α-(1,3)-glucan mediates Treg response whereas the shorter α-(1,3)-oligosaccharide efficiently blocked this Treg activation^38^. It could be speculated that, in our experimental conditions, conidia are recognized by receptors responsible of their anchorage to the epithelial cell membranes and that favors the encounter of conidia *via* their FleA lectin with another specific receptor. As a result, each conidia adhere concomitantly to epithelial cells through “anchorage” receptors and specific “FleA” receptors. Such a binding of conidia to clusters of receptors with possibly increased avidity, would prevent their germination. A such inhibition is supported by the results of Torosantucci *et al*. demonstrating that anti-β-glucan antibodies bound to *A*. *fumigatus* inhibit filament growth *in vitro* in the absence of immune-effector cells^39^. The authors hypothesized that anti-β-glucan antibodies may have direct fungal inhibitory activity through interactions with crucial fungal molecules. Interestingly, the hypothesis of cluster formation also explains the results with recombinant FleA. Thus, although conidia are still “anchored” to cells, the occupation of the “FleA” receptor by the recombinant lectin prevents the cluster formation and consequently the antifungal activity. Of note, inhibition of PI3-kinase would inhibit the expression of either one of these receptors.

To the best of our knowledge, the current report presents the first evidence of a direct and strong fungistatic activity of bronchial epithelial cells against *A*. *fumigatus*. Our results show that epithelial antifungal activity is PI3-kinase dependent and involves the lectin FleA. We demonstrated that in addition to a role in the inflammatory response, FleA functions as an adhesin and that its recognition by epithelial cells contributes to the control of *A*. *fumigatus* infection. As lectins interact with proteins and carbohydrate moieties expressed by host cells, the next step would be to identify the receptor expressed by epithelial cells that binds to the lectin FleA. To this end, we will perform a transcriptomic analysis of bronchial epithelial cells incubated with or without PI3 kinase inhibitors and infected with *A*. *fumigatus*. Thus, genes coding receptors significantly differentially expressed will be selected and then knockdown one after the other using specific siRNAs. The correlation between the reduced expression of a mRNA and its corresponding protein and increased conidia germination will then be evaluated. Understanding the detailed mechanism of the interactions between the host and *A*. *fumigatus* may ultimately contribute to improving the treatment of patients.

## Materials and Methods

### Reagents

F-12 nutrient mixture, penicillin and streptomycin, glutamine, and trypsin-EDTA were purchased from Life Technologies (Carlsbad, CA, USA). The P38 MAPK (SB203580), PI3-kinase (LY294002, Wortmannin), and ERK1/2 (PD98059) inhibitors were from Cell Signaling Technology (Danvers, MA, USA). Primary human airway epithelial cells (hAEBCs) were commercially purchased from Epithelix Sarl (Plan-les-Ouates, Switzerland) and their use does not require ethical approval. The Platelia Aspergillus EIA kit was obtained from Bio-Rad (Marnes la Coquette, France). The human bronchial epithelial cell line, BEAS-2B, was obtained from the American Type Cell Collection (Manassas, VA, USA). Fluorescein isothiocyanate (FITC) was purchased from Sigma-Aldrich (Saint-Louis, MO, USA) and anti-FITC rabbit antibody was from Invitrogen (Carlsbad, CA, USA). Corning Transwell insert (0.4 µm) filters were obtained from Sigma-Aldrich (Saint-Louis, MO, USA). Chemicals used in FleA expression and purification were bought from Fischer Scientific (Illkirch, France) or Sigma-Aldrich (Saint-Quentin-Fallavier, France).

### Preparation of *A*. *fumigatus* conidia

*A*. *fumigatus* DAL strain (CBS 144.89), initially isolated from a human patient with invasive aspergillosis in France, was obtained from J.P. Latgé (Laboratoire des Aspergillus, Institut Pasteur - Paris, France)^40^. *A*. *fumigatus* clinical strains were isolated as part of the routine mycological surveillance in patients suffering from COPD or CF and sent to us anonymously. All strains were maintained on 2% malt agar for 1 week at 27 °C. Conidia were harvested from the slant into 0.1% Tween-20 in phosphate-buffered saline (PBS). The resulting suspensions were centrifuged for 10 min at 10,000 × *g*, the pellet was resuspended in PBS/Tween, and the number of conidia per mL was determined with a hemocytometer. The concentration was adjusted at 10^3^ conidia in a total volume of 300 μL of culture medium.

### Lectin preparation

The nucleotide sequence of FleA was amplified from the published plasmid^16^ using the PrimeSTAR Max DNA Polymerase (Takara Bio Europe), 5′-catccatggctactcctggagcacagcaagtc-3′ as forward primer and 5′-cgctcgagttaagcaggaggaagagcac-3′ as reverse primer following the manufacturer’s instructions. After digesting the PCR product with the *Nco*I and *Xho*I restriction enzymes, the gene was introduced in the expression vector pET32b-TEV using those restriction sites. This homemade vector is derived from the pET32b plasmid (Novagen, Madison WI) where the enterokinase cleavage site was replaced for the cleavage site of the Tobacco Etch virus (TEV) protease. *Escherichia coli* BL21 (DE3) cells harboring the plasmid pET32b-TEV-FleA were cultured in LB medium with 100 µg mL^−1^ ampicillin at 37 °C. When the culture reached an absorption of 0.6 at 595 nm (A595nm), the incubation temperature was modified to 16 °C. When A595nm reached 0.8–0.9, protein expression was induced by adding 0.1 mM isopropyl β-D-thiogalactoside (Euromedex, Souffelweyersheim, France). Cells were harvested after one night at 16 °C by centrifugation at 5000 × *g* for 5 min at 4 °C. Each gram of cell pellet from 1 L culture medium was resuspended in 5 mL buffer A (20 mM Tris-HCl pH 8, 500 mM NaCl, and 20 mM imidazole-HCl pH 8) and 1 µL benzonase (Sigma-Aldrich, Saint-Quentin-Fallavier, France) was added. After a 15-min incubation at room temperature, cells were disrupted at a pressure of 1.9 kBar (Constant Cell Disruption System) and centrifuged (22 000 × *g*, 30 min, 4 °C). The cell lysates were filtered using a 0.45-µM syringe filter (Dutscher, Issy les Moulineaux, France) and loaded onto a 1-ml HisTrap^TM^ column (GE Healthcare, France) equilibrated with buffer A for affinity chromatography. After washing with buffer A, elution was performed with a 20-mL linear gradient of imidazole (0–500 mM). The fractions were analyzed on 12% SDS-PAGE gels prior to pooling and desalting on a PD10 column (GE Healthcare) in 20 mM Tris-HCl, pH 8 and 100 mM NaCl. The fusion was cleaved with the TEV protease^41^ (1:50 w/w; enzyme:protein ratio) at 19 °C overnight after addition of 0.5 mM EDTA and 0.5 mM TCEP. The mixture was applied on a HisTrap column and the cleaved FleA protein was collected in the flow-through. Finally, FleA was concentrated by centrifugation using a Vivaspin (3 kDa, Sartorius Stedim Biotech) to the desired concentration and stored at 4 °C. This recombinant lectin FleA maintains an active conformation as shown by its binding to fucose and fucosylated glycoconjugates with high affinity (16).

### Cell culture and stimulation

Human bronchial epithelial BEAS-2B cells were maintained and serially passaged in F-12 culture medium supplemented with 10% fetal calf serum (FCS), 1% penicillin and streptomycin, and 10 mM HEPES in 75-cm^2^ culture flasks, and they were seeded (5 × 10^4^ cells) into 24-well plates 3 days before stimulation. In all experiments, BEAS-2B cells were stimulated for 15 h with conidia (10^3^) in 300 μL of medium, without penicillin and streptomycin, corresponding to a 0.001 MOI. In some cases, cells were pre-incubated with kinase inhibitors, such as LY294002 (30 µM) and Wortmannin (10 µM) targeting PI3-kinase, SB203580 (30 µM) and PD98059 (30 µM) targeting P38 MAPK and ERK1/2, respectively, or with dimethyl sulfoxide (DMSO), the solvent, for 1 h before the infection. Inhibitors and the solvent stayed in the culture medium during the 15-h incubation with *A*. *fumigatus*.

To test the role of the lectin, FleA, cells were pre-incubated with different concentrations of lectin (0.1, 0.5, and 1 µM) in the presence or absence of 1 mM oligosaccharide (methyl-α-L-fucopyranoside or D-galactose). After 1 h, cells were infected with 10^3^ conidia without removing the lectin and incubated for 15 h. To study the effect of FleA on conidia adhesion, 10^3^ conidia were added to BEAS-2B cells incubated for 1 h with 1 µM FleA. After 1, 2, 4, and 6 h, the supernatants were collected, cells were washed with 300 µL of the medium to remove the non-adherent conidia. For each well, supernatant and washing were pooled in the same tube, 100 µL was plated on Petri dishes, and incubated overnight at 37 °C. The CFU were counted to determine the number of non-adherent conidia and then the number of adherent conidia was calculated.

Commercial primary human airway epithelial cells from bronchial biopsies (hAEBCs) were received at passage 1 and cultured in 75-cm^2^ culture flasks in serum-free Epithelix hAEC culture medium, which was changed every 3 days. One week later, cells were subcultured in 24-well plates. After reaching confluence, they were incubated for 15 h with conidia (10^3^) in 300 μL hAEC medium.

To evaluate the cellular antifungal activity, the conidia were incubated in parallel under the same conditions (concentration, culture medium, and the well size) without epithelial cells.

### Microscopic score and galactomannan measurement

Following 15 h of incubation, conidia (10^3^ per well) were microscopically observed for scoring the formation of filaments. Presence of filaments was scored from 1 (no filament) to 6 (maximum level of filament formation) (arbitrary units). The reference value, scored 3, was determined as the filament formation on the cultured epithelial cells without inhibitors. For each experimental condition, three wells were used per set of experiments, and three independent blind observers scored microscopic observations.

Galactomannan is a polysaccharide released by growing *A*. *fumigatus* and used as an index of filament formation. Galactomannan antigen concentrations were determined by using a commercially available kit, Platelia Aspergillus EIA. To quantify galactomannan, supernatants were treated as recommended by the manufacturer for serum treatment. Values were expressed as a percentage of the values for the control, i.e., *A*. *fumigatus* incubated without cells.

### Impact of the antifungal activity on the viability of the fungus

BEAS-2B cells were infected with 100 conidia for 6 h. This low inoculum was used on purpose for CFU counting to avoid further dilutions that could have introduced a bias in the counting. Aliquots of the inoculum were plated on a 2% malt-extract agar Petri dish to control the number of conidia incubated with cells at T0. Afterward, at 6 h after the infection, supernatants were collected, and the epithelial cells were lysed with water. Lysates and supernatants were pooled to recover all conidia and centrifuged. The pellets were re-suspended, plated on Petri dishes, and incubated overnight at 37 °C. CFU numbers were determined to confirm the viability of the conidia.

### Screening for a released antifungal component

Using Transwells, BEAS-2B cells were cultured in wells until they reached confluence. Inserts with permeable membrane (0.4 µm pore size), permitting diffusion of media components, were placed in plate wells with or without confluent cell monolayers. Conidia (10^3^) were added to the inserts for 15 h. Then, filament formation was scored (arbitrary units as described above) under the microscope and galactomannan measured in the supernatant.

### Conidial internalization and index of germination

Conidia were labeled with FITC (0.1 mg/mL in 0.05 M Na_2_CO_3_ buffer, pH 9) overnight at 4 °C and washed with PBS/Tween 0.1%. Epithelial cells were infected with 10^3^ FITC-labeled conidia for 6 h in the presence or absence of the PI3-kinase inhibitor LY294002 (30 µM). Following this, the medium was removed, and the cells were fixed with 3% paraformaldehyde, blocked with PBS and 1% bovine serum albumin, and incubated with an anti-FITC rabbit antibody for 1 h. The cells were washed prior to the incubation with a secondary anti-rabbit antibody labeled with AlexaFluor 568. Cell nuclei were stained with DAPI. Preparations were observed by using an epifluorescence microscope. Intracellular conidia appeared green, whereas the extracellular conidia, accessible to the antibody labeled with red AlexaFluor 568, appeared orange (green and red superposed). This labeling allowed us to determine the role of PI3-kinase in internalization of conidia by epithelial cells. A hundred resting or germinated conidia per well were counted, and the percentage of internalized conidia relative to the total number of conidia was expressed in the presence or absence of LY294002. This labeling was also used to determine the role of PI3-kinase in the inhibition of germination of extracellular or intracellular conidia by counting only germinated conidia. We determined the percentage of extracellular germinated conidia and intracellular germinated conidia, relative to the total number of conidia, in the absence or presence of LY294002.

### Statistics

Data are presented as the mean value ± standard error of mean (SEM). Statistic tests were performed using Prism 6.00 software (GraphPad Software, La Jolla, CA). Differences between two groups were tested using the unpaired *t*-test. ANOVA was performed to compare quantitative variables across groups. To correct for multiple testing, we used the Bonferroni’s method (comparisons of selected groups); p < 0.05 was considered statistically significant. Correlation between microscopic score and galactomannan measurement was obtained by using the Pearson’s test.
